# Effectiveness and economic impact of interventions targeting healthcare personnel: a systematic review of return on investment

**DOI:** 10.3389/phrs.2026.1609191

**Published:** 2026-08-10

**Authors:** Pierluigi Catalfo, Chiara Cantarella, Daniele Virgillito, Helena C. Maltezou, Caterina Ledda

**Affiliations:** 1 Economic and Business Department, University of Catania, Catania, Italy; 2 Clinical and Experimental Medicine Department, University of Catania, Catania, Italy; 3 National Public Health Organization, Athens, Greece

**Keywords:** economic impact, healthcare personnel, preventive healthcare, professional development, return on investment (ROI)

## Abstract

**Objectives:**

To evaluate and critically interpret economic evidence on workplace interventions targeting healthcare personnel, distinguishing return on investment from related economic evaluation approaches and grouping interventions by underlying mechanisms.

**Methods:**

PubMed, Scopus, and Web of Science were searched for peer-reviewed studies published from 2006 to 2024. Eligible empirical studies reported return on investment, benefit-cost ratios, net savings, cost-effectiveness metrics, societal returns, or comparable monetized outcomes for workplace interventions involving healthcare workers. Methodological quality was assessed using CHEERS and ECOBIAS.

**Results:**

Twenty-eight studies met the inclusion criteria. Most reported positive or potentially favorable economic returns, particularly for preventive health measures, workplace mental health and disability-management programs, and educational or human-capital initiatives. Findings were highly context-dependent. Some interventions produced modest, neutral, or unfavorable returns, while the highest estimates often came from model-based analyses or broad societal extrapolations. Comparability was limited by differences in perspective, time horizon, monetization assumptions, health-system structure, labor costs, and implementation.

**Conclusion:**

Evidence supports workplace interventions as part of health workforce strategies, but return on investment should not be used as a stand-alone decision criterion. Future evaluations require standardized methods, transparent assumptions, and explicit perspectives and time horizons.

## Introduction

Healthcare personnel (HCP) face numerous challenges in their daily work environments, including burnout, stress, and occupational diseases. Burnout, characterized by emotional exhaustion, depersonalization, and reduced personal accomplishment, is prevalent among HCP [1, 2]. Factors contributing to burnout include high workload, role ambiguity, and moral distress from inability to provide optimal care [3, 4]. Stress of conscience, arising from conflicts between professional duties and personal values, significantly impacts burnout [1]. Work-related stress affects physical and mental health, leading to increased absenteeism [5]. Personality traits like negative affectivity can exacerbate the effects of workplace stressors [6]. Addressing these challenges requires systemic changes, including improved work organization, professional supervision, and opportunities for reflection [4, 7]. Implementing evidence-based interventions and promoting a healthy work environment are crucial for mitigating burnout and its consequences [7].

Workplace interventions addressing wellbeing, safety, and professional development are crucial for improving employee outcomes and organizational performance. Research shows that learning interventions can positively impact wellbeing [8], while leadership development can enhance employee health and safety [9]. Total Worker Health® interventions, combining occupational safety and health with wellness, effectively reduce risk factors for injuries and chronic illnesses [10]. Workplace resources at individual, group, leader, and organizational levels contribute to both employee wellbeing and performance [11]. However, interventions for health and social service workers often show modest, short-term effects due to barriers such as high workload and lack of support [12–14]. A comprehensive approach to stress management and wellbeing interventions, tailored to specific organizational needs, is recommended for sustained improvements in employee health and organizational effectiveness [15–17].

Research indicates that initiatives focusing on HCP wellbeing, safety, and training can positively impact performance and retention. Well-designed health facilities can improve working conditions, staff safety, and efficiency [18]. Implementing wellbeing programs can increase workplace engagement, job satisfaction, and organizational performance [19, 20]. A comprehensive approach to promoting a safety culture and addressing physical and psychological hazards, can improve both HCP and patient safety [21]. Evidence-based institutional programs and individual interventions can reduce burnout and improve wellbeing [22]. Retention strategies include leadership support, professional development, recognition, improved work environment, and flexible scheduling [23]. Overall wellbeing is a significant predictor of healthcare costs, productivity, and retention outcomes [24]. However, more research is needed to evaluate the effectiveness of these initiatives [23, 25].

HCP safety and preparedness significantly impact healthcare system efficiency and outcomes. Inadequate infection prevention and control measures can lead to HCP infections, compromising patient care and system resilience [26]. Well-designed healthcare interventions can improve staff safety, job satisfaction, and performance [18]. Organizational factors, work environment, and culture also affect worker health and patient outcomes [27]. During outbreaks, HCP face increased risks, with infection rates ranging from 1% to 57% for various emerging infectious diseases [28]. Prioritizing preparedness and implementing rapid risk assessments can mitigate these risks [26, 29]. Factors influencing HCP’ willingness to respond during crises include the nature of the event, competing obligations, work environment, and perceived efficacy [30]. Addressing these issues is crucial for maintaining a robust healthcare system and ensuring quality patient care [31].

Return on Investment (ROI) is a widely used metric for evaluating workplace interventions, but its application and interpretation vary. While some studies report positive ROI for interventions targeting work-family conflict [32], obesity [33], and sedentary behavior [34], others caution against overreliance on ROI [35–37]. For instance, Cherniack [35] notes that ROI can be overestimated and may discount long-term health benefits. Thonon et al. [36] found that most studies report positive ROI, but randomized controlled trials show fewer positive results. Grant [37] argues that ROI is insufficient for measuring coaching outcomes and proposes alternative frameworks. To address these limitations, researchers have developed more comprehensive approaches, such as the systems approach [38] and the use of ROI in conjunction with other metrics to evaluate human capital investments [39]. These approaches aim to provide a more holistic assessment of workplace interventions.

In detail, measuring ROI for healthcare programs related to safety, wellbeing, and professional development, can provide evidence for their cost-effectiveness. Studies have shown that ROI analysis can guide resource allocation decisions for professional development activities [40, 41]. ROI calculations have been used to demonstrate the financial impact of nursing professional development programs [42] and unit-based health promotion activities [43, 44]. In the context of safe patient handling and mobility programs, ROI models can estimate potential economic benefits in intensive care units [45]. Leadership development programs have also been evaluated using ROI indicators to assess their impact on healthcare organizations [46]. Recent updates in ROI measurement techniques for professional development activities emphasize the importance of linking educational interventions to economic outcomes [47, 48], providing a comprehensive approach to evaluating program effectiveness.

Despite growing research on workplace interventions for HCP, the economic evidence remains difficult to synthesize because interventions, outcomes, evaluation perspectives, and healthcare financing contexts vary substantially. This review therefore uses a conceptual framework to distinguish intervention mechanisms and economic evaluation approaches before interpreting the findings. The aim is not to collapse all economic evaluations into a single ROI metric, but to examine how different forms of monetized evidence have been used to justify or assess investments in healthcare personnel.

First, ROI is interpreted as a financial ratio comparing the monetized net benefits of an intervention with its costs. Cost-benefit analysis, cost-effectiveness analysis, cost-utility analysis, and SROI were treated as related but conceptually distinct approaches. CBA and NPV/IRR estimates are grounded in economic valuation of costs and benefits; CEA and ICERs compare additional costs with additional health or productivity outcomes; SROI attempts to monetize broader social value. In the synthesis, these approaches were coded separately and discussed according to the perspective adopted by the original authors, rather than being treated as interchangeable forms of ROI [49–52].

Second, the review differentiates between employer or institutional perspective and societal perspective. Employer-perspective analyses usually emphasize fiscal savings, productivity gains, absenteeism reduction, revenue recovery, or training returns accruing to an organization. Societal-perspective analyses may incorporate avoided mortality, population health gains, broader economic productivity, or social value, and therefore tend to produce larger estimates that are not directly comparable with institution-level ROI.

Third, the review distinguishes short-term fiscal savings from long-term human-capital investment. Some interventions generate rapid cost offsets, such as vaccination or PPE during outbreaks, whereas professional development, onboarding, research awards, or workforce-retention programs may require longer time horizons before returns become visible. This distinction was used to interpret both positive and neutral findings and to avoid overstating the policy transferability of single-study ROI values.

**FIGURE 1:**
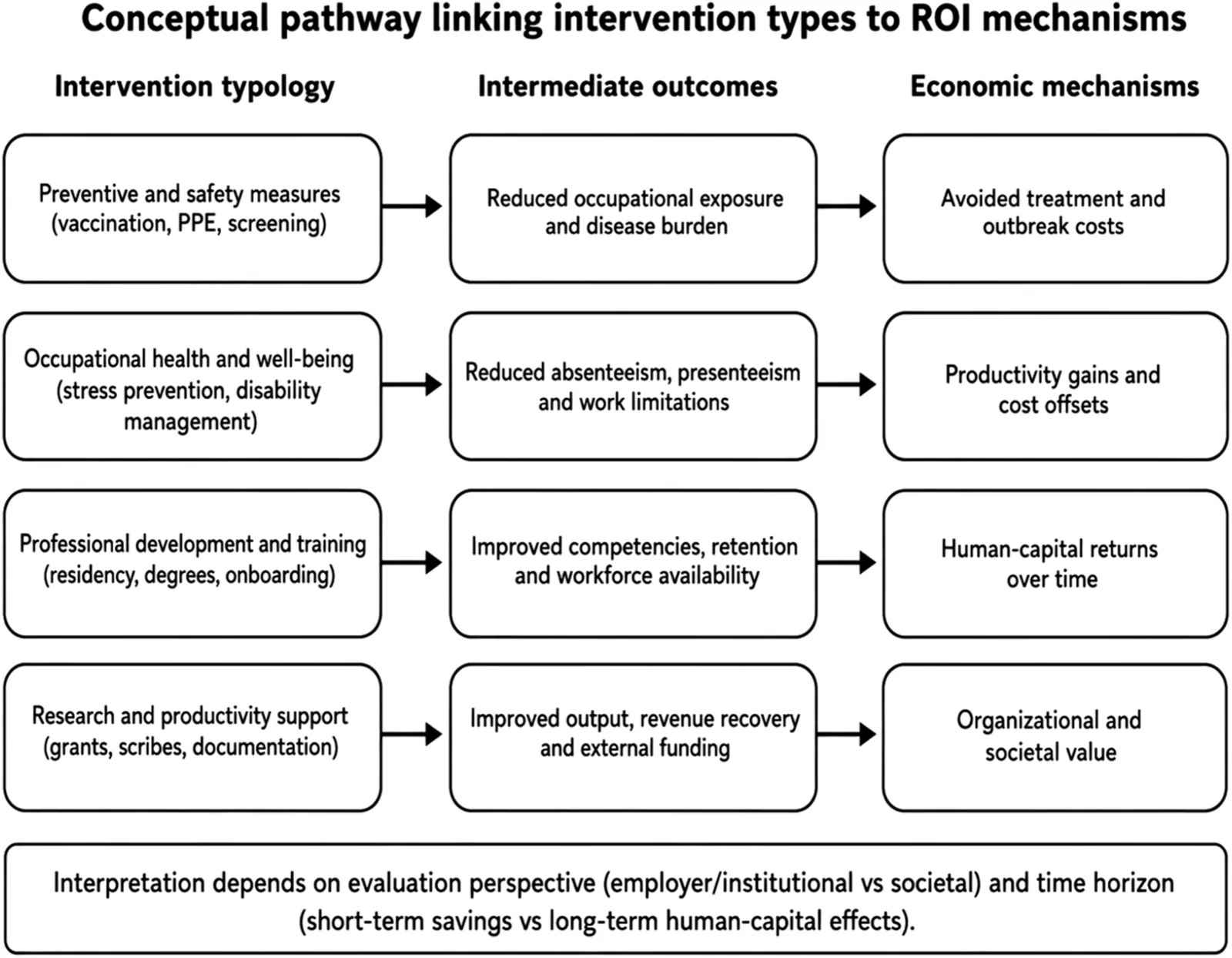
Conceptual pathway linking workplace intervention types for healthcare personnel to intermediate outcomes and economic mechanisms relevant to return on investment. The figure shows how preventive, occupational health, training, and productivity-support interventions may generate economic value through reduced disease burden, lower absenteeism and presenteeism, improved retention and competencies, and increased organizational or societal value. Interpretation depends on the evaluation perspective and time horizon adopted.

The objective of this systematic review is therefore to assess the economic impact of workplace interventions targeting HCP while explicitly accounting for intervention typology, evaluation perspective, time horizon, and the distinction between ROI and adjacent economic evaluation methods. Figure 1 summarizes the conceptual pathway used to interpret the included evidence.

## Methods

### Study design and protocol

A systematic review was conducted to assess the effectiveness and economic impact of workplace interventions targeting HCP, with a specific focus on interventions reporting a measurable economic return or monetized outcome. Studies were included if they evaluated interventions aimed at burnout prevention, wellbeing, safety, professional development, workforce retention, or organizational performance and reported ROI, net savings, benefit-cost ratios, SROI, cost-effectiveness ratios, or comparable human-capital metrics.

The research question was developed using the PICO framework, which considers Population, Intervention, Comparison, and Outcome. As outlined in Table 1, this framework was used to guide the systematic review process and to ensure a structured definition of the key components of the research question [50]. The framework facilitated the identification of eligible studies, categorization of interventions, and evaluation of outcomes related to ROI and adjacent economic evaluation metrics.

**TABLE 1:** PICO framework used to define the review question and eligibility criteria. The table summarizes the population, intervention, comparison, and outcome elements used to identify studies evaluating the economic value and return on investment of workplace interventions for healthcare personnel.

Element	Description
P (Population)	Healthcare personnel (HCP), including physicians, nurses, and allied health workers in hospital and healthcare settings
I (Intervention)	Workplace interventions aimed at improving burnout, wellbeing, safety, professional development, or retention of HCP.
C (Comparison)	No intervention or different types of workplace interventions (e.g., burnout prevention vs. professional development programs)
O (Outcome)	Return on investment (ROI), measured through cost savings, productivity improvements, retention rates, reduced absenteeism, improved employee wellbeing, and organizational performance

By applying this PICO framework, the review systematically identified and analyzed studies that evaluated interventions for HCP with a measurable economic outcome. This allowed for a comprehensive understanding of the financial and non-financial benefits associated with workplace strategies targeting HCP wellbeing, safety, professional development, and organizational performance.

The review adhered to the PRISMA (Preferred Reporting Items for Systematic Reviews and Meta-Analyses) guidelines to ensure rigorous methodology and transparency throughout the selection process [53, 54].

A comprehensive search was conducted on January 24, 2025, across three electronic databases: PubMed, Scopus, and ISI/Web of Science. The search strategy involved a combination of keywords and Medical Subject Headings (MeSH) terms relevant to the target population, interventions, and outcomes. The following keywords were used: “healthcare personnel”, “workplace intervention”, “burnout”, “wellbeing”, “safety”, “professional development”, “return on investment”, “ROI”, “healthcare workers”, “employee retention”, and “organizational performance”. Boolean operators such as AND, OR, and parentheses were used to refine the search and ensure the inclusion of studies that met the predefined eligibility criteria.

Only peer-reviewed published studies were included. Grey literature, unpublished reports, conference abstracts without full empirical data, and internal institutional documents were excluded to ensure source comparability, reproducibility of data extraction, and alignment with the journal requirement that reviews refer only to published data. This choice may have reduced the risk of including non-peer-reviewed economic estimates, but it may also have excluded implementation reports with negative or null findings.

### Study selection process and eligibility criteria

After the initial retrieval of search results, citations from peer-reviewed articles were extracted from the primary databases. To streamline the elimination of duplicates and support the management of the articles selected for inclusion, Rayyan software was used [55]. Three independent reviewers (P.C., C.C, and C.L.) were involved in the selection process.

The screening was carried out in two phases. In the first phase, two reviewers (P.C. and C.L.) independently assessed the titles, abstracts, and keywords of the articles to evaluate their relevance to the scope of the review. Studies not meeting the inclusion criteria were excluded. Relevant review articles were also identified for potential citation searching to uncover additional studies not indexed in the selected databases [56].

In the second phase, the same reviewers evaluated the full-text articles according to the inclusion and exclusion criteria. Any disagreements were resolved through consultation with the third reviewer (C.C.), who oversaw the process and ensured consistency across both phases.

Eligible studies for this systematic review were required to meet the following criteria: (1) studies that focused on HCP, including physicians, nurses, and allied health workers, and involved workplace interventions targeting their wellbeing, safety, professional development, retention, or organizational performance; (2) studies that reported ROI or a monetized economic outcome as a primary or secondary outcome, including direct and indirect cost measures, benefit-cost ratios, net savings, SROI, ICERs, or human-capital returns; (3) studies published in peer-reviewed journals; (4) studies that included a control group, a comparator, a pre- and post-intervention design, or an explicit economic model; and (5) studies published between 2005 and 2024 in English.

Studies were excluded from this review if they: (1) did not report ROI or a comparable quantitative economic outcome; (2) provided only anecdotal or qualitative data without quantifiable economic results; (3) involved interventions that did not directly target HCP or the healthcare workforce; or (4) were opinion articles, editorials, conference abstracts, unpublished reports, grey literature, or case reports lacking a clear research methodology or empirical data.

### Quality assessment

The quality of reporting and the potential risks of bias were evaluated for all studies that included economic evaluations, using the Consolidated Health Economic Evaluation Reporting Standards 2022 [57] and the Risk of Bias in Model-based Economic Evaluations (ECOBIAS) checklist. CHEERS 2022 is the updated version of the original CHEERS checklist, which first appeared in 2013 with 24 items [48, 57]. The 2022 version expands to 28 items, providing guidance to healthcare stakeholders and ensuring standardized reporting of health economic evaluations. The additional four items in CHEERS 2022 include the health economic analysis plan, characterizing distributional effects, and evaluating the effect of such engagement. ECOBIAS, consisting of a 22-item checklist, assists researchers in evaluating the risk of bias in both model-based and trial-based economic evaluations, focusing on the methodological aspects of the studies [58].

### Definitions

To ensure terminological consistency and enhance the interpretability of findings across diverse study contexts, the following key definitions were adopted and applied uniformly throughout this systematic review.

- HCP: Refers to all individuals engaged in the delivery of healthcare services, either directly (e.g., physicians, nurses, midwives) or indirectly (e.g., laboratory technicians, pharmacists, and allied health professionals). This definition aligns with the World Health Organization’s global health workforce classification [59].
- ROI: A key economic metric representing the ratio between the net benefit of an intervention and its associated cost. In healthcare, ROI encompasses both direct savings (e.g., reduced absenteeism or turnover) and indirect gains (e.g., increased productivity or quality of care) [50].
- Workplace Intervention: Any organized program, policy, or action implemented within a healthcare setting with the intent to enhance employee wellbeing, safety, efficiency, or retention. This includes interventions focused on physical health, mental health, professional development, and organizational culture [16].
- Preventive Healthcare Measure: A proactive strategy aimed at averting illness, injury, or occupational health risks. Examples include vaccination campaigns, routine health screenings, or ergonomic modifications in the workplace [60].
- Professional Development Program: Structured educational or training activities aimed at improving the skills, knowledge, and career trajectories of HCP. These may include continuing education courses, residencies, mentorship programs, or certification pathways [47].
- Occupational Health Outcome: Any physical, psychological, or psychosocial health consequence arising from workplace exposures, job demands, or organizational conditions. Such outcomes include stress, burnout, musculoskeletal injuries, and work-related illness [61].
- Cost-Effectiveness: An economic evaluation framework comparing the relative costs and health outcomes of two or more interventions. It is widely used in healthcare to support resource allocation decisions, especially under budget constraints [49].

Cost-Benefit Analysis (CBA), Net Present Value (NPV), and Internal Rate of Return (IRR): Economic valuation approaches that monetize costs and benefits or discount future cash flows to estimate whether an investment produces a net financial gain. In this review, these were interpreted as financial/economic evaluation methods rather than as synonymous with ROI.

Societal Return on Investment (SROI): An approach that attempts to monetize broader social, organizational, or population-level value. SROI may capture benefits beyond the employer perspective, but its results are highly sensitive to valuation assumptions and should not be directly compared with narrow institutional ROI estimates.

Perspective and time horizon: Perspective refers to the stakeholder whose costs and benefits are counted, such as the employer, healthcare institution, payer, taxpayer, or society. Time horizon refers to the period over which costs and benefits are measured. Both were extracted where reported because they strongly influence the magnitude and interpretation of ROI.

## Results

### Literature search

**FIGURE 2:**
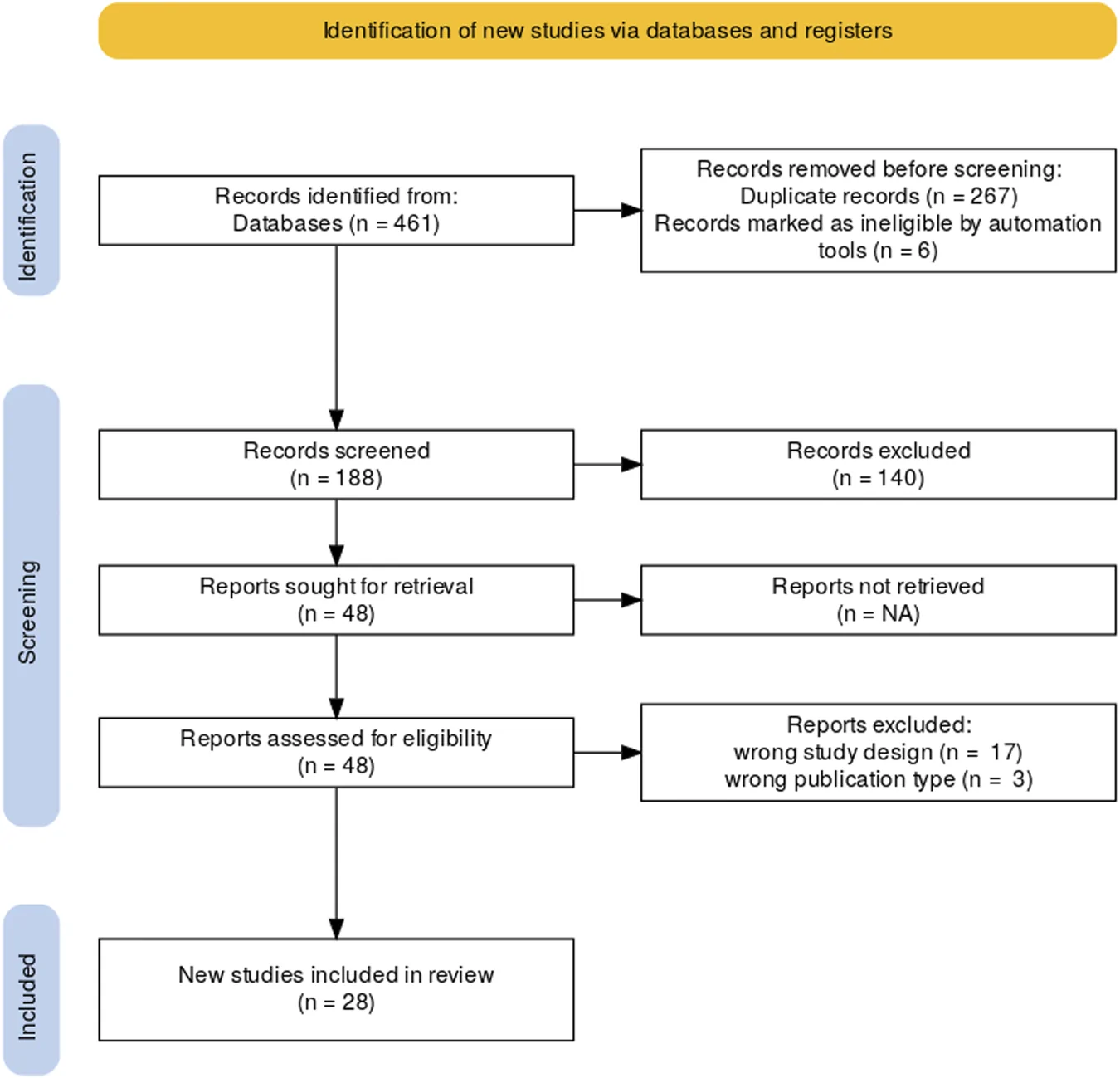
PRISMA flow diagram showing the identification, screening, eligibility assessment, and inclusion process for studies evaluating the economic impact of workplace interventions among healthcare personnel.

A comprehensive search across several electronic databases initially yielded 644 records. The PRISMA flowchart (Figure 2) details the process of selecting relevant publications at each step of the review. After eliminating 86 duplicate entries, 558 records advanced to the screening phase. During this stage, 475 records were excluded based on their failure to meet the established inclusion criteria. This left 83 articles to be further assessed for eligibility. In the final phase, 28 studies were included in the qualitative synthesis.

### General characteristics of the included studies

This systematic review includes 28 studies that examine the effects of workplace interventions on HCP. The studies analyzed both direct and indirect outcomes related to ROI [62–89] (Table 2).

**TABLE 2:** Characteristics of the 28 studies included in the systematic review. The table summarizes key study features, including author, year, country or setting, intervention type, target population, economic evaluation approach, and main economic outcomes. Source: authors’ synthesis of the 28 studies included in this review (2006–2023).

Study	Year	Country	Study design	Population	Intervention type	Comparison	Outcome measures	ROI estimation	Key findings	CHEERS quality assessment	ECOBIAS quality assessment
[62]	2006	Canada	Cost-benefit analysis	International medical graduates in Alberta (AIMG)	Skills assessment and residency training	Before and after AIMG program implementation	Financial rate of return to taxpayers, residency readiness, employment as physicians	Internal Rate of Return (IRR) calculated	AIMG program yields 9%-13% annual rates of return; cost-effective compared to expanding medical school capacity	High	Medium
[63]	2006	US	Program evaluation	Investigators at the University of Minnesota Academic Health Center	Seed grant program and interdisciplinary/intercollegiate Faculty Research Development (FRD) grant program	Before and after grant program implementation	External grants, peer-reviewed publications	ROI calculated based on subsequent external grants and publications	Seed grant program yielded 560% ROI; FRD grant program yielded 237% ROI; successful in generating external funds and publications, stimulating risk-taking, and fostering collaboration	High	Medium
[64]	2006	US	Longitudinal repeated measures design	590 underserved men	Outreach by community health workers (CHWs)	9 months before and after interaction with a CHW	Service utilization, charges, reimbursements, uncompensated costs	ROI calculated based on changes in service utilization and costs	ROI of 2.28:1.00; annual savings of $95,941; increased primary and specialty care visits; decreased urgent care, inpatient, and outpatient behavioral healthcare utilization	Medium	Medium
[65]	2007	Malawi	Cost analysis	Medical doctors in Malawi	Migration of medical doctors	Before and after migration	Cost of training, lost investment returns	Future Value (FV) calculated based on interest rates	Cost of training one doctor: $56,946.79; lost investment returns over 30 years: $433,493 to $46 million depending on interest rates	Medium	High
[66]	2010	US	Retrospective observational study	Medical students pursuing careers in internal medicine, psychiatry, gastroenterology, general surgery, or orthopedics	Military vs. civilian medical careers	Military vs. civilian career paths over 30 years	Net present value (NPV) of educational investments	NPV calculated using a 5% discount rate	In the civilian sector, the lowest return on educational investment was for psychiatrists ($1.136 million) and the highest for orthopedists ($2.489 million). In the military, the lowest returns were for internists ($1.377 million) and the highest for orthopedists ($1.604 million). The range of returns in the military ($0.227 million) was one-sixth that in the civilian sector ($1.354 million). Most military physicians do not remain in the military for their full careers.	High	High
[67]	2010	US	Retrospective cost-benefit analysis	Pharmacy residents at Bay Pines Veterans Affairs Healthcare System	Pharmacy residency training program	Before and after residency program implementation	Resident productivity, cost savings, training costs	ROI calculated based on cost-benefit analysis	Benefit-to-cost ratio of 1.5:1; annual savings of $563,936; resident productivity estimated at 62.8% of preceptor output	High	High
[68]	2011	Sub-Saharan Africa (Ethiopia, Kenya, Malawi, Nigeria, South Africa, Tanzania, Uganda, Zambia, Zimbabwe)	Human capital cost analysis	Doctors from nine sub-Saharan African countries	Emigration of doctors to Australia, Canada, the UK, and the USA	Before and after emigration	Financial cost of educating doctors, lost investment returns	Lost returns on investment calculated using compounding interest	Estimated loss of $2.17bn from emigration; South Africa and Zimbabwe had the greatest economic losses; destination countries saved $4.55bn in training costs	High	High
[69]	2013	Netherlands	Randomized controlled trial	730 older hospital workers	Worksite vitality intervention (yoga, aerobic exercise, coaching, fruit)	Usual care	General vitality, work-related vitality, need for recovery, costs	Incremental cost-effectiveness ratios, ROI from employer's perspective	No significant differences in costs and effects; incremental cost-effectiveness ratios: €280 (general vitality), €7506 (work-related vitality), €258 (need for recovery); ROI: €2.21 lost per euro invested	Medium	Medium
[70]	2013	Netherlands	Randomized controlled trial	163 temporary agency and unemployed workers	Participatory return-to-work (RTW) program	Usual care	Sustainable RTW, quality-adjusted life years (QALY), costs	Incremental cost-effectiveness ratio (ICER), net societal benefit	Participatory RTW program was more effective but more costly; ICER: €80 per day earlier RTW; net societal benefit: €2073 per worker; ROI: 0.89	Medium	High
[71]	2015	Netherlands	Cluster-randomized trial	413 nurses in a Dutch academic hospital	Preventive intervention (screening, feedback, referral to occupational physician)	Screening without feedback and unrestricted access to usual care	Absenteeism, presenteeism, intervention costs, productivity	ROI calculated based on cost offsets from reduced absenteeism and presenteeism	Net savings of €244 per nurse (absenteeism only) and €651 per nurse (absenteeism and presenteeism); ROI of €5 to €11 per euro invested	High	High
[72]	2015	Netherlands	Economic model analysis	Healthcare workers (HCWs) in a neonatology ward	Preventive vaccination against pertussis	No vaccination	Nosocomial pertussis outbreak costs, vaccination costs	ROI calculated based on averted outbreak costs	ROI of 1:4, meaning 4 Euros saved for every Euro invested; total outbreak costs: €48,682; vaccination costs: €12,208	High	Medium
[73]	2016	US	Markov modeling and break-even analysis	High school graduates, bachelor's degree holders, pharmacy graduates	Obtaining a PharmD degree	High school diploma, bachelor's degree in biology or chemistry	Net cumulative income, break-even point	ROI calculated based on net cumulative earnings	Pharmacy graduates surpass net cumulative earnings of high school graduates by age 33 and bachelor's degree holders by age 34; net earnings of $716,345 to $1,064,840 over the first 10 years of a pharmacy career	High	High
[74]	2016	US	Retrospective analysis	Trauma surgeons at a level 1 trauma center	Focused documentation improvement (FDI)	Pre-FDI documentation	Case mix index (CMI), revenue recovery, physician response rate	ROI calculated based on revenue recovery	CMI increased from 1.80 to 2.11 (p < 0.001); revenue recovery of $1,132,581; ROI of 220%	High	High
[75]	2017	US	Economic analysis	Physician assistants (PAs) and nurse practitioners (NPs)	Obtaining a PA or NP degree	RN with a 4-year degree	Net present value (NPV), return on investment (ROI)	ROI calculated based on NPV	PAs have a slightly higher ROI than NPs; NPV of $781,323 for PAs and $764,348 for NPs; NPV of $728,436 for RNs with a 4-year degree	High	Medium
[76]	2017	Australia	Cost analysis	Health professionals across 12 disciplines	Training programs	Not applicable	Cost of training per year of practice	Variable ROI based on retention rates	General medical practitioners: $18,432 per year; Dentists: $11,158 per year; Dieticians: $10,277 per year; Audiologists: $10,304 per year; Podiatrists: $5,170 per year; Optometrists: $4,870 per year	High	High
[77]	2018	US	Productivity and revenue analysis	Academic urology practice	Medical scribes	Pre-scribe period	Productivity, revenue, and satisfaction	Return-to-investment ratio greater than 6:1	Increased efficiency and work satisfaction; 2.15 more patients per session; additional 2.6 wRVUs; $542 in physician charges; $861 in hospital charges per session; net financial impact of $429 per session	High	High
[78]	2020	Italy	Retrospective observational study	Healthcare workers (HCWs) in a pediatric hospital	Workplace Disability Management Program (WDMP)	Pre-WDMP period	Absenteeism, productivity, and limitations	ROI of €27.66 for each euro invested	Significant reduction in absenteeism and work limitations; total savings of €127,896 over a year; break-even point reached with 327 HCWs	High	High
[79]	2020	South Africa	Economic analysis	Healthcare professionals (HCPs) supported by the Umthombo Youth Development Foundation (UYDF)	Tertiary education funding	Not applicable	Costs and economic value of training HCPs	Internal rate of return of 63%	Total cost of training 254 graduates was ZAR186 million; graduates expected to generate ZAR15 billion in lifetime earnings; income tax on future earnings estimated at ZAR4 billion	High	Medium
[80]	2020	Low- and middle-income countries (LMICs)	Cost-effectiveness and ROI analysis	Healthcare workers (HCWs)	Personal protective equipment (PPE)	No PPE intervention	Mortality, cost per HCW case averted, cost per HCW life saved	ROI of $755.3 billion USD (7,932% return)	Investment of $9.6 billion USD would save 2,299,543 lives; cost of $59 USD per HCW case averted and $4,309 USD per HCW life saved	High	High
[81]	2020	Netherlands	Matched cohort study in two parallel groups	Healthcare workers (mainly nurses)	Stress-Prevention@Work implementation strategy	Waitlisted control condition	Productivity losses, absenteeism, presenteeism	ROI of 59.6 (close to 60-fold payout per euro invested)	Net benefit of €2981 per employee per year; 96.7% likelihood of breaking even within 1 year; 98.2% likelihood of net benefit of at least €1000	High	High
[82]	2022	Italy	Pilot study	Healthcare workers (HCWs)	Psychological support programme	Control group of 245 workers	Occupational distress, quality of life, sickness absence	ROI of 2.73	Significant reduction in distress and improvement in quality of life; 60% reduction in sickness absence days; ROI of 2.73	Medium	High
[83]	2021	Kenya	Cost-effectiveness and ROI analysis	Healthcare workers (HCWs)	Personal protective equipment (PPE)	No PPE intervention	Incremental cost per HCW death averted, incremental cost per HCW COVID-19 case averted	ROI of $170.64 million (11.04 times return)	Investment of $3.12 million would avert 416 HCW deaths and 30,041 HCW COVID-19 cases; ROI of 11.04 times the investment	High	High
[84]	2021	Lao PDR	Cost-effectiveness analysis	Healthcare workers (HCWs)	Seasonal influenza vaccination	No vaccination	Incremental cost per life-year saved (ICER), incremental cost per HCW COVID-19 case averted	ROI of 2.88 Kip for every single Kip invested	Vaccination could save 1,393 doctor visits, 81 days of hospitalizations, 9,453 days of work annually; net cost of five billion Kip (599,391 USD) annually	High	Medium
[85]	2022	Italy	Cost-benefit analysis	Healthcare workers (HCWs) at Fondazione Policlinico Universitario Agostino Gemelli IRCCS	COVID-19 vaccination campaign	No vaccination	Costs and benefits associated with the vaccination campaign	Benefit-to-cost ratio (BCR) of EUR 4.66; societal return on investment (SROI) of EUR 3.66	Vaccination campaign costs: EUR 2,221,768; benefits: EUR 10,345,847; prevented long absences from work and adverse financial, staffing, and managerial consequences	High	High
[86]	2023	Italy	Econometric analysis	Healthcare workers (HCWs)	Stop Smoking Program (SSP)	Before and after SSP intervention	Sickness Absence Days (SADs), ROI, break-even analysis	ROI of 1.90; break-even point of 7.85	SSP intervention showed a success rate of 37.5% after 2 years; significant reduction in absenteeism (85% reduction in 1 year); nurses and physicians had the highest success rate	High	High
[87]	2023	US	Pre/post intervention study	Newly hired nurse practitioners (NPs) and physician assistants (PAs)	Structured onboarding program	Before and after onboarding program	Presurveys/postsurveys, attrition rates, onboarding costs	Not explicitly calculated, but cost savings noted	Structured onboarding program reduced attrition from 10.3% to 4.5%; annual cost for onboarding participants was $63,470 versus $256,826 as the estimated mean cost of one separation within their first year	High	Medium
[88]	2023	US	Survey and database analysis	Neurosurgeons	Neurosurgery Research and Education Foundation (NREF) awards	Before and after NREF funding	Subsequent grant funding, academic achievements	NREF impact ratio: $1:$134	NREF funding led to significant subsequent grant funding ($776M); highest ROI for Young Clinician Investigator (YCI) awards ($1:$381); 73% of awardees in academic positions; 82% received subsequent research grants	High	High
[89]	2023	US	Descriptive study	Surgeons (residents and junior faculty)	AAS/SUS research awards	Not applicable	Scholarly achievements, NIH funding, academic positions	79-fold ROI for residents; 98-fold ROI for junior faculty	High success in academic surgery; 39% of resident awardees and 50% of junior faculty awardees obtained NIH funding; many hold leadership positions (professors, division chiefs, department chairs)	High	High

The included studies span a period of 17 years, ranging from 2006 to 2023. The temporal distribution reveals a growing interest in evaluating the ROI of workplace interventions targeting HCP. The included studies were conducted across diverse geographical regions, reflecting a broad global interest in assessing the ROI of workplace interventions for healthcare personnel. The geographical distribution of the studies reveals a significant concentration in North America, with the United States accounting for 39.29% of the total studies. This is followed by the Netherlands, which contributes 17.86% of the studies, and Italy, with 14.29%. Other countries represented include Canada, Malawi, sub-Saharan African countries, Australia, South Africa, Kenya, and Lao PDR, each contributing 3.57% of the studies. Additionally, one study encompasses multiple low- and middle-income countries (LMICs), also representing 3.57% of the total. This distribution highlights the predominance of research conducted in high-income countries, particularly the United States, while also reflecting a diverse range of international contexts.

For interpretability, studies were also considered by income setting and analytical perspective. High-income country studies more often reported employer, institutional, educational, or revenue-related outcomes, whereas LMIC or multi-country analyses more frequently used model-based estimates of avoided mortality, infection, or macroeconomic productivity losses. These differences informed the narrative synthesis and caution against direct cross-country comparison of ROI values.

The analysis of study designs reveals a diverse array of methodologies employed across the research. Cost-benefit analysis and cost analysis each account for 7.14% of the studies, reflecting a strong focus on evaluating the financial implications of healthcare interventions. Similarly, retrospective observational studies and randomized controlled trials also represent 7.14% each, highlighting the importance of both observational and experimental approaches in healthcare research. Other notable study designs include economic analysis and cost-effectiveness and ROI analysis, each contributing 7.14% to the total. This variety in study designs underscores the multifaceted nature of healthcare research, which encompasses program evaluations, longitudinal studies, and various economic models. The presence of unique designs such as human capital cost analysis, Markov modeling, and break-even analysis further illustrates the innovative approaches researchers employ to assess the impact of healthcare programs. The distribution of study designs demonstrates a balanced use of both qualitative and quantitative methods, providing a comprehensive understanding of the economic and practical outcomes of healthcare interventions.

### Results of characteristics referred to PICO

The analysis of the populations studied reveals a diverse range of groups, reflecting the broad scope and inclusivity of healthcare research. The most frequently studied population is general HCP, who are the focus of 28.57% of the total studies. This significant representation underscores the critical importance of understanding the impact of healthcare interventions on those directly involved in patient care, particularly in light of recent global health challenges such as the COVID-19 pandemic [90]. The emphasis on HCP highlights the need to support and protect this essential workforce through various programs and interventions.

To address the substantial heterogeneity of interventions, the synthesis grouped interventions according to their dominant mechanism of action rather than treating all ROI estimates as directly comparable. Table 3 presents the typology used in the review, including examples, primary economic mechanisms, and interpretive cautions.

**TABLE 3:** Typology of workplace intervention mechanisms and return-on-investment pathways. The table groups the included interventions into four categories and summarizes how each may generate economic value, including avoided disease and replacement costs, reduced absenteeism and presenteeism, improved retention and workforce productivity, and broader organizational or societal returns. Typology developed by the authors based on synthesis of the 28 studies included in this systematic review, covering multiple countries and the period 2006–2023.

Intervention typology and ROI pathway
Preventive and safety measures. Examples include vaccination campaigns, PPE provision, infection prevention and screening. The main economic pathway is avoided disease, outbreak containment, reduced sick leave and avoided replacement costs. These estimates are often highly context-sensitive and, when based on modelling, should be interpreted in relation to local incidence, unit costs, vaccine/PPE prices and healthcare-system capacity
Occupational health, wellbeing and return-to-work interventions. Examples include stress-prevention programmes, worksite vitality interventions, screening with referral to occupational physicians, and disability-management or return-to-work programmes. The main pathway is reduced absenteeism and presenteeism, earlier return to work, improved retention and productivity. Implementation intensity, adherence, baseline risk and follow-up duration strongly influence ROI.
Professional development and human-capital interventions. Examples include residency training, PharmD, physician assistant or nurse practitioner education, and skills assessment programmes. The pathway is long-term human-capital accumulation, productivity, workforce supply and career earnings. These studies require attention to time horizon, discounting, retention, migration and whether the perspective is individual, institutional or societal
Research, documentation and productivity interventions. Examples include seed grants, research awards and focused documentation improvement. The pathway is external grant capture, revenue recovery, publications or efficiency gains. ROI can be large but may depend on attribution assumptions, selection effects and the distinction between financial return and broader academic or societal value

Medical graduates, including international medical graduates, medical physicians, and medical students pursuing various specialties, account for 14.29% of the studies. These groups are crucial for understanding the broader implications of healthcare training and migration, as well as the economic returns on investment in medical education and professional development. For instance, the study on international medical graduates in Alberta, Canada provides insights into the financial benefits of skills assessment and residency training programs [62], while the research on medical doctors in Malawi highlights the economic impact of doctor migration on healthcare systems in sub-Saharan Africa [65].

Surgeons and surgical professionals, including trauma surgeons, neurosurgeons, and surgical residents, also represent 10.71% of the studies. These studies provide valuable insights into the specific challenges and economic implications of surgical training and practice [88].

Pharmacy professionals, including pharmacy residents and graduates, represent 7.14% of the studies. These populations are essential for evaluating the effectiveness of educational and training programs in preparing HCP for their roles [63].

Nurses and nurse practitioners, who account for 10.71% of the studies, are another critical group. Research on this population focuses on workplace interventions and preventive measures aimed at improving health and productivity. For instance, the study on nurses in a Dutch academic hospital examines the impact of preventive interventions on absenteeism and presenteeism [71], highlighting the economic benefits of investing in the wellbeing of HCP.

Finally, other populations, such as investigators, underserved men, older hospital workers, and temporary agency workers, account for 28.57% of the studies. These diverse groups highlight the wide-ranging nature of healthcare research, which aims to address the needs and challenges of various segments of the population. By examining a broad array of populations, these studies provide valuable insights into the effectiveness and economic implications of healthcare programs across different contexts.

Four intervention categories were identified. Preventive and safety measures included vaccination programs, PPE provision, screening, and occupational-health referral pathways. These interventions generally generated returns through avoided infections, reduced outbreak costs, lower absenteeism, and prevention of work-related functional limitations.

Occupational health and wellbeing interventions included worksite vitality programs, stress-prevention implementation strategies, workplace disability management, psychological support, and smoking-cessation programs. The worksite vitality intervention, for example, combined yoga, aerobic exercise, coaching, and fruit provision. These programs were mainly linked to reduced absenteeism and presenteeism, improved vitality, lower need for recovery, and productivity gains, although some trials reported limited or unfavorable economic returns.

Professional development and human-capital investments included residency training, advanced degrees, onboarding programs, tertiary education funding, and professional training. Their ROI mechanisms were longer-term and included increased earnings, workforce retention, resident or staff productivity, reduced turnover, and the availability of qualified HCP.

Research, documentation, and service-productivity interventions included seed grants, research awards, focused documentation improvement, and medical scribes. These interventions generated returns through subsequent grant funding, academic outputs, revenue recovery, improved documentation quality, or increased clinical productivity. Because their outcomes differ substantially from health and wellbeing outcomes, their ROI estimates were interpreted within this specific organizational-productivity category.

The analysis of comparison types reveals a diverse range of approaches used to evaluate the effectiveness of healthcare interventions. The most frequently used comparison types are “no vaccination” and “not applicable,” each accounting for 10.71% of the studies [63, 72, 84, 85, 89]. The “no vaccination” comparison is particularly relevant in studies assessing the impact of vaccination programs, such as those for pertussis, seasonal influenza, and COVID-19. These comparisons highlight the importance of preventive measures in reducing disease incidence and improving public health outcomes. The “not applicable” category includes studies where a direct comparison was not feasible or necessary, such as those focusing on the economic analysis of training programs or the impact of research awards. This category underscores the variety of methodologies employed in healthcare research, where some studies rely on economic modeling or descriptive analysis rather than direct comparisons.

Other common comparison types include “usual care” (7.14%) and “no PPE intervention” (7.14%). The “usual care” comparison is used in studies evaluating the effectiveness of new interventions against standard practices, such as worksite vitality interventions and return-to-work programs. The “no PPE intervention” comparison is relevant in studies assessing the impact of PPE on HCP safety and infection rates [69, 70, 83].

Several studies use a “before and after” comparison, examining the impact of interventions over time. Examples include “before and after Alberta International Medical Graduate program implementation,” “before and after grant program implementation,” and “before and after migration.” These comparisons, each representing 3.57% of the studies, are valuable for understanding the longitudinal effects of healthcare programs and policies [62, 63, 65].

The distribution of comparison types demonstrates the comprehensive approach taken in healthcare research to evaluate the effectiveness and economic implications of various interventions. By employing a wide array of comparison methods, these studies provide robust evidence to inform healthcare policies and practices, ultimately aiming to improve health outcomes and support the sustainability of healthcare systems worldwide.

The analysis of outcome measures reveals a diverse range of metrics used to evaluate the effectiveness and impact of healthcare interventions. Each outcome measure is represented by 3.57% of the studies, except for absenteeism, presenteeism, intervention costs, and productivity, which account for 7.14% of the studies. This variety in outcome measures underscores the multifaceted nature of healthcare research, which aims to capture both economic and health-related impacts.

Financial metrics, such as the financial rate of return to taxpayers, net present value (NPV) of educational investments, and cost of training, are commonly used to assess the economic benefits of healthcare programs. These measures provide insights into the financial returns on investment in healthcare education and training, highlighting the long-term economic value of such initiatives [66].

Productivity and cost-related measures, including resident productivity, cost savings, training costs, and productivity losses, are also frequently used. These metrics emphasize the importance of efficiency and cost-effectiveness in healthcare interventions, demonstrating how investments in training and preventive measures can lead to significant cost savings and productivity gains [73, 74].

Health-related outcomes, such as service utilization, quality-adjusted life years (QALY), and mortality, are critical for evaluating the direct impact of healthcare interventions on patient health and wellbeing. For example, studies on vaccination programs and PPE use measure the reduction in disease incidence and mortality, underscoring the importance of preventive healthcare measures [80, 83, 84, 91].

Additionally, measures of scholarly and academic achievements, such as external grants, peer-reviewed publications, and subsequent grant funding, highlight the role of research and education in advancing medical knowledge and practice [88, 89]. These outcomes demonstrate the success of grant programs and research awards in fostering academic excellence and collaboration.

The distribution of outcome measures reflects the comprehensive approach taken in healthcare research to evaluate the effectiveness and economic implications of various interventions. By employing a wide array of metrics, these studies provide robust evidence to inform healthcare policies and practices, ultimately aiming to improve health outcomes and support the sustainability of healthcare systems worldwide.

### Return on investment and key results

Economic findings were synthesized by distinguishing direct ROI estimates from adjacent economic evaluation approaches. Direct ROI, benefit-cost ratios, net savings, revenue recovery, and productivity gains were interpreted as financial-return measures. NPV, IRR, and CBA were interpreted as investment appraisal or welfare-based economic valuation approaches. ICERs and cost-effectiveness ratios were not treated as ROI, but as complementary evidence on whether additional costs were justified by additional outcomes. SROI and human-capital estimates were interpreted separately because they incorporate broader societal or long-term value.

This distinction is important because the magnitude and meaning of economic returns depend strongly on the method used. For example, IRR and NPV estimates in educational or workforce-investment studies capture discounted financial returns over long time horizons, whereas employer-perspective ROI studies on absenteeism or productivity capture short-to medium-term fiscal savings. Similarly, ICERs provide information on value for money but do not necessarily indicate a positive financial return to an employer.

Several studies reported direct cost offsets through revenue recovery, reduced absenteeism, or productivity gains. Focused documentation improvement and medical-scribe interventions were primarily linked to revenue or productivity mechanisms, while preventive occupational health programs were linked to reduced absenteeism and presenteeism. These findings suggest plausible financial value, but they remain dependent on local reimbursement rules, wage structures, baseline absenteeism rates, and implementation costs.

Exceptionally high ROI figures require particular caution. Estimates such as the $755.3 billion USD value and 7,932% return reported for PPE in LMICs, or other large returns for PPE and stress-prevention strategies, were driven by model-based assumptions, broad societal extrapolations, avoided mortality, and scenario parameters. These values are important for illustrating the potential scale of prevention benefits, but they should not be interpreted as directly transferable institutional budget returns.

The review also identified modest, neutral, or unfavorable findings. In the worksite vitality RCT, no significant differences in costs and effects were observed and the employer-perspective ROI indicated a loss per euro invested. The participatory return-to-work intervention was more effective but more costly, with an ROI below 1. These findings underscore that economic value depends on implementation fidelity, baseline risk, uptake, time horizon, and the perspective adopted by the analysis.

Overall, the included studies provide evidence that many workforce-targeted interventions can generate economic value, but this conclusion is strongest when interventions are interpreted within comparable categories and perspectives. The synthesis therefore emphasizes patterns and mechanisms rather than pooled or directly ranked ROI values.

One of the significant findings is the high ROI for many healthcare interventions. For instance, the study by Emery et al. [62] on the Alberta International Medical Graduate (AIMG) program in Canada found that the program yields annual rates of return between 9% and 13%, making it a cost-effective alternative to expanding medical school capacity. Similarly, Paller et al. [63] reported a 560% ROI for a seed grant program and a 237% ROI for an interdisciplinary Faculty Research Development (FRD) grant program in the US, demonstrating the success of these programs in generating external funds and publications.

Several studies emphasize the economic benefits of preventive interventions. For example, Tariq et al. [72] found that preventive vaccination against pertussis for HCP resulted in a 4:1 ROI, meaning that every euro invested in vaccination saved four euros in outbreak costs. Ortega-Sanchez et al. [84] reported a 2.88 Kip ROI for seasonal influenza vaccination, highlighting the cost savings associated with preventing influenza cases among HCP. Nurchis et al. [85] found that a COVID-19 vaccination campaign at Fondazione Policlinico Universitario Agostino Gemelli IRCCS in Italy yielded a benefit-to-cost ratio (BCR) of 4.66 and a societal return on investment (SROI) of 3.66, underscoring the financial and societal benefits of vaccination programs.

Workplace interventions also show significant economic returns. Noben et al. [71], in a study conducted in the Netherlands, reported that a preventive intervention involving screening, feedback, and referral to occupational physicians resulted in net savings of €244 to €651 per nurse, with an ROI of €5 to €11 per euro invested. Camisa et al. [78] found that a Workplace Disability Management Program for HCP in a pediatric hospital in Italy yielded an ROI of 27.66€ for each euro invested, demonstrating the program’s effectiveness in reducing absenteeism and work limitations.

The studies also highlight the importance of educational and training programs in generating economic returns. Chisholm-Burns et al. [73], in the US, found that pharmacy graduates surpass the net cumulative earnings of high school graduates by the age of 33 years and bachelor’s degree holders by the age of 34 years, with net earnings ranging from $716,345 to $1,064,840 over the first 10 years of a pharmacy career. Craig et al. [75] reported that physician assistants (PAs) and nurse practitioners (NPs) have a slightly higher ROI than registered nurses (RNs) with a 4-year degree, emphasizing the financial benefits of advanced healthcare education.

These findings underscore that structured educational and training investments—whether clinical, academic, or research-based—play a pivotal role in advancing workforce competencies and generating long-term financial returns for both individuals and institutions.

Research awards and grant programs also demonstrate substantial economic and academic benefits. Javeed et al. [88] found that Neurosurgery Research and Education Foundation (NREF) awards in the United Kingdom led to significant subsequent grant funding, with an NREF impact ratio of $1:$134. Olutoye et al. [89] reported a 79-fold ROI for residents and a 98-fold ROI for junior faculty who received AAS/SUS research awards, highlighting the success of these awards in fostering academic excellence and securing NIH funding.

Overall, the key findings from these studies illustrate the diverse benefits of healthcare interventions, ranging from financial returns to improvements in health outcomes and productivity. These findings provide robust evidence to inform healthcare policies and practices, ultimately aiming to improve health outcomes and support the sustainability of healthcare systems worldwide.

### Quality assessment

The quality assessments of the studies, based on the CHEERS and ECOBIAS criteria, reveal a high standard of research across the majority of the studies. According to the CHEERS criteria, 82.14% of the studies are rated as high quality, while 17.86% are rated as medium quality. This indicates that most studies adhere to rigorous reporting standards, ensuring transparency and comprehensiveness in the presentation of health economic evaluations.

The ECOBIAS quality assessment further supports these findings, with 67.86% of the studies rated as high quality and 32.14% as medium quality. The ECOBIAS criteria focus on the potential biases in economic evaluations, assessing the methodological rigor and the extent to which studies minimize bias in their analyses. The high percentage of studies rated as high quality suggests that the majority of the research employs robust methodologies, reducing the risk of bias and enhancing the reliability of the findings.

These quality assessments are crucial for interpreting the results of the studies and for making informed decisions based on their findings. High-quality studies provide more reliable evidence, which is essential for policymakers, healthcare providers, and other stakeholders who rely on this research to guide healthcare policies and practices. The adherence to CHEERS and ECOBIAS standards ensures that the studies are not only methodologically sound but also transparent in their reporting, allowing for critical appraisal and replication of the research.

## Discussion

The findings from this systematic review provide a structured and critical synthesis of economic evidence on workplace interventions targeting HCP. The results should not be interpreted as proof that all interventions generate generalizable positive ROI. Rather, they indicate that economic returns are most plausible when interventions have a clearly defined mechanism, when the relevant perspective is explicit, and when costs and benefits are measured over an appropriate time horizon.

Preventive and safety interventions, such as vaccination and PPE, tended to generate value through avoided infections, outbreak costs, absenteeism, and mortality. Occupational health and wellbeing interventions generated value mainly through reduced absenteeism, presenteeism, work limitations, and improved productivity. Professional development and training programs generated longer-term returns through human-capital accumulation, retention, and workforce capacity. Research and productivity interventions generated value through revenue recovery, academic outputs, or external funding.

ROI analysis in healthcare settings is complex and multifaceted. While traditional financial methods can provide insights into specific projects, they often fail to capture the full impact of healthcare Information Technology (IT) investments [92]. Econometric approaches have shown promise in demonstrating the positive effects of IT on hospital productivity and quality [92]. ROI calculations in healthcare vary widely, with some focusing solely on fiscal savings and others incorporating broader benefits like monetized health outcomes [51]. Studies have found significant returns on healthcare investments, with estimates ranging from $1.10 to $38.00 for every dollar spent, depending on the specific intervention [93, 94]. To improve consistency and interpretability, researchers recommend standardized methodologies and reporting guidelines for ROI analyses in healthcare [51]. Mobile health clinics have also demonstrated substantial ROI, with one study reporting a 36:1 return [95].

Healthcare interventions can have significant long-term economic impacts on organizational performance. Positive psychology interventions can improve wellbeing and support among HCP, potentially enhancing organizational performance [96]. A four-year follow-up study demonstrated sustained improvements in wellbeing at work for HCP following a targeted intervention [97]. Long-term reductions in adverse psychosocial factors and improvements in health indicators were observed 3 years post-intervention in an acute care hospital [98]. Healthcare innovations generally show positive impacts on organizational performance, particularly in health utilization metrics and medical-clinical indicators [99]. Fact-based psychosocial workplace interventions can enhance employee wellbeing and organizational performance, including increased productivity and decreased absenteeism [100]. However, interventions may have unintended consequences, necessitating a multi-stakeholder, multivariate longitudinal perspective when assessing their financial impact [101].

ROI analysis is increasingly used to evaluate healthcare interventions, but methodologies and definitions vary widely ([51, 52]. While some studies focus solely on fiscal savings, others incorporate broader benefits like monetized health outcomes [51]. ROI in healthcare quality improvement is often conceptualized as value or benefit for stakeholders, rather than purely financial returns [52]. Studies have found positive ROI for various interventions, including workplace wellness programs [102, 103], obesity management [102], and overall healthcare spending [93]. However, ROI estimates can be influenced by factors such as program length, company size, and study quality [103]. Some researchers advocate for more sophisticated statistical techniques and consideration of non-linear relationships in ROI calculations [104]. Recent models have also incorporated patient access alongside traditional cost and quality metrics [105].

Preventive healthcare interventions consistently demonstrate high ROI across various settings. A systematic review found a median ROI of 14.3:1 for public health interventions, with national programs yielding even higher returns [106, 107]. Workplace-based prevention programs also show positive ROIs in 56.5% of cases [36]. Specific interventions with high ROIs include complex case management (12.21:1), asthma management (6.35:1), and diabetes care (1.16:1) [107–109]. A comprehensive wellness program demonstrated sustained economic value over 8 years, with an average ROI of 2.02:1 [110]. Factors contributing to high ROIs include targeting high-risk populations, addressing conditions associated with avoidable emergency and inpatient utilization, and implementing interventions at a national level [107–109]. These findings suggest that investing in preventive healthcare is cost-effective and can generate substantial savings.

Workplace health programs in the healthcare sector can positively influence employee productivity and wellbeing. Studies have shown that comprehensive wellness initiatives can reduce health risks, absenteeism, and work-related exhaustion while improving employee engagement, job satisfaction, and overall productivity [100, 111, 112]. Mental health support programs, including employee assistance programs and stress management workshops, contribute to enhanced wellbeing and increased engagement [113, 114]. Perceived workplace health support is associated with higher work productivity, particularly in reducing presenteeism [115]. Successful programs often involve strong leadership participation, employee involvement, and integration into organizational culture [111]. However, some studies have found limited causal effects on medical expenditures and health behaviors [116]. Despite this, workplace wellness programs generally demonstrate a positive return on investment and can lead to improvements in employee health, productivity, and organizational performance [117–119].

Effective healthcare education and training programs are crucial for improving patient care and organizational performance. Key determinants of success include learner-centered approaches, relevance to daily practice, and adequate duration [120, 121]. These programs can yield positive ROI [122] by increasing revenue, reducing absenteeism, and improving work performance [86, 122, 123]. Successful programs often involve interdisciplinary teamwork, experienced facilitators, and the use of e-learning resources [120]. Evaluating program effectiveness requires comprehensive assessment frameworks that measure organizational excellence and healthcare outcomes [46, 124]. Despite their importance, many private healthcare facilities fail to provide adequate education and training, particularly in waste management [125]. Overall, investing in well-designed healthcare education and training programs can lead to significant improvements in patient care, staff performance, and organizational success.

Research on healthcare interventions reveals significant differences in outcomes between high-income countries (HICs) and LMICs. Cancer mortality-to-incidence ratios are higher in LMICs, with WHO healthcare scores correlating with improved outcomes [126]. Traumatic brain injury studies show higher mortality rates in LMICs for severe cases, but lower disability rates for mild and moderate cases [127]. Economic evaluations differ across income groups, reflecting variations in healthcare capacity and research expectations [128]. Public health interventions generally demonstrate high cost-effectiveness, with median ROI of 14.3:1 overall and 27.2:1 for nationwide interventions in HICs [106]. However, ROI analyses vary in methodology and included benefits, necessitating careful interpretation for policy decisions [51]. These findings highlight the need for context-specific research and adaptation of interventions across different income settings.

In the present review, global applicability is constrained by the predominance of high-income settings. HIC studies generally used employer, institutional, educational, or revenue perspectives, while LMIC studies were more likely to use model-based analyses of PPE, avoided mortality, or system-level protection of the health workforce. For this reason, ROI values should be interpreted within income-setting strata and should not be transferred across countries without adjustment for wages, workforce shortages, financing arrangements, procurement costs, and baseline service capacity.

Healthcare interventions like vaccination programs targeting HCP and workplace health initiatives contribute significantly to healthcare system sustainability. Vaccination reduces healthcare costs by preventing diseases and associated complications, making it one of the most cost-effective interventions [129, 130]. Workplace health initiatives, such as enforcing occupational safety regulations and providing flexible working arrangements, can positively impact workers’ health and job sustainability [131]. However, sustainability of healthcare interventions faces challenges, including inconsistent definitions and measures [132]. The nature of interventions influences their sustainability, requiring tailored strategies for different types of programs [133, 134]. A health system approach can improve immunization program performance and strengthen overall healthcare systems [135]. To enhance sustainability, researchers suggest adopting a dynamic framework that allows for continuous adaptation and improvement of interventions in changing contexts [136–138].

ROI is increasingly used to evaluate healthcare interventions, but its application has both strengths and limitations. ROI can provide valuable information on the economic benefits of public health programs [51, 139]. However, the methodology and definition of ROI in healthcare vary widely, leading to inconsistencies in calculations and reporting [51, 52]. ROI often focuses on short-term financial outcomes, potentially overlooking long-term health benefits and non-monetary outcomes [35, 140]. The metric may be influenced by various institutional factors, making it challenging to balance different stakeholder interests [140]. While ROI can be a useful tool for decision-making, it should be interpreted cautiously and not used as the sole criterion for resource allocation in healthcare [141, 142]. A more comprehensive approach that considers both monetary and non-monetary benefits may be more appropriate for evaluating healthcare quality improvement initiatives [140].

A further implication is that ROI should not dominate decisions about occupational health investments. Critical health economics literature warns that ROI may undervalue intangible benefits such as dignity, psychological wellbeing, fairness, safety culture, and long-term workforce resilience, while overemphasizing short-term monetizable gains [140, 141]. Therefore, ROI is best used as one component of a broader decision framework that also includes ethical, clinical, organizational, and equity considerations.

This review presents several notable strengths that enhance the credibility and relevance of its findings. First, it is one of the few systematic reviews to specifically focus on the economic outcomes of workplace interventions targeted at HCP, an area of increasing importance given the global workforce shortages and rising healthcare expenditures. Second, the inclusion of a broad range of interventions, spanning from PPE and vaccination campaigns to residency training, workplace disability programs, and research grants, offers a comprehensive overview of strategies adopted across diverse healthcare systems. This heterogeneity allows for the comparison of different models of investment in HCP across contexts, disciplines, and professional categories. Third, the review covers multiple countries and healthcare settings, including acute care hospitals, trauma centers, academic medical institutions, and occupational health services. This diversity enhances the applicability of findings across various organizational environments. Fourth, economic evaluation metrics were systematically extracted and discussed, including ROI, cost-benefit analyses, cost-effectiveness ratios, and productivity gains. By doing so, the review not only identifies effective interventions but also provides quantitative evidence to inform financial planning and policy decisions in the healthcare sector. Finally, the review integrates findings from studies conducted before, during, and after health crises, offering unique insights into how economic outcomes may shift depending on external stressors such as pandemics. This temporal depth enriches the discussion and supports more adaptive and resilient health workforce strategies.

While this systematic review provides useful insights into the economic impact of workplace interventions targeting HCP, several limitations must be acknowledged when interpreting the findings.

First, generalizability is limited by substantial heterogeneity in intervention type, target population, healthcare system, labor market, and evaluation perspective. ROI estimates reported in high-income settings may not be directly applicable to LMICs, where labor costs, procurement conditions, workforce shortages, and healthcare infrastructure differ substantially. Conversely, societal or model-based estimates from LMICs should not be interpreted as equivalent to employer-level budget returns in high-income systems.

Second, the temporal context of the studies may influence results. Some interventions were evaluated before the COVID-19 pandemic and before recent shifts in workforce shortages, inflation, digitalization, and health-system resilience planning. Economic returns from infection-prevention or preparedness interventions may change considerably during future outbreaks or emergencies.

Third, many included studies lacked standardization in ROI calculation methods, perspectives, and time horizons. The heterogeneity in how benefits such as productivity, cost savings, avoided mortality, academic outputs, and wellbeing were monetized prevented pooled quantitative synthesis. Fourth, the exclusion of grey literature and the predominance of positive ROI findings raise the possibility of publication bias, as null or negative implementation experiences may be underreported. Future studies should report assumptions transparently, include sensitivity analyses, and clearly distinguish ROI, CBA, CEA, SROI, and human-capital approaches.

This systematic review underscores the importance of targeted interventions in enhancing the wellbeing, safety, professional development, and sustainability of HCP. The revised synthesis suggests that policy recommendations should be intervention-specific rather than based on undifferentiated ROI values.

For preventive and safety interventions, decision makers should prioritize settings with high baseline risk, clear exposure pathways, and measurable avoided costs. For occupational health and wellbeing interventions, expected ROI should be assessed alongside implementation fidelity, workforce participation, organizational support, and sustainability. For professional development and training programs, evaluation horizons should be sufficiently long to capture retention, skill acquisition, and human-capital returns.

For all intervention categories, future evaluations should report the analytical perspective, time horizon, costing method, monetization assumptions, sensitivity analyses, and distributional or equity implications. CHEERS 2022 and ECOBIAS offer useful reporting and bias-appraisal standards, but additional workforce-specific guidance may be needed to capture intangible outcomes such as safety culture, psychological wellbeing, and professional development.

Overall, the results support evidence-based investment in HCP, but they also reinforce the need for careful interpretation of ROI. Economic returns should be considered together with ethical obligations to protect workers, patient safety, workforce resilience, and the long-term sustainability of healthcare systems.
